# Spherical Reverse Beamforming for Sound Source Localization Based on the Inverse Method

**DOI:** 10.3390/s19112618

**Published:** 2019-06-09

**Authors:** Chao Sun, Yuechan Liu

**Affiliations:** 1 School of Measurement and Communication Engineering, Harbin University of Science and Technology, Harbin 150080, China; sc13579@126.com; 2 Post-Doctor Research Center of Power Engineering and Engineering Thermophysics, Harbin Engineering University, Harbin 150001, China; 3 College of Underwater Acoustic Engineering, Harbin Engineering University, Harbin 150001, China

**Keywords:** spherical reverse beamforming, inverse problem, *p*-norm constraint, sound source localization

## Abstract

A spherical array is not limited to providing an acoustic map in all directions by the azimuth of the array. In this paper, spherical reverse beamforming for sound source localization based on spherical harmonic beamforming and the principle of sound field reconstruction is proposed in order to output a sharper scanning beam. It is assumed that there is an imaginary sound source at each scan point, and the acoustic map of a spherical array to the actual sound source is regarded as the combination of all of the imaginary sound sources. Sound source localization can be realized by calculating the contribution of each imaginary sound source to the sound field. Also in this work, the non-convex constrained optimization problem is established using *p*-norm. Combined with the norm method, the sparse solution of the imaginary sources is obtained through iterative weighted techniques, and the resolution of sound source localization is improved significantly. The performance of this method is investigated in comparison to conventional spherical beamforming. The numerical results show that the proposed method can achieve higher resolution for the localization of sound sources without being limited by the frequency and array aperture, and has a stronger ability to suppress fluctuations in background noise.

## 1. Introduction

The focused beamforming array signal processing technique is widely applied to radiation noise detection and the positioning of aircrafts, automobiles and ships due to its good tolerance and simplicity [1]. However, the conventional linear array tends to have a blind scanning area. The spherical array can be widely used for sound field analysis and sound source localization in three-dimensional space due to its symmetry and rotation.

In spherical array beamforming, which was first presented by Meyer and Abhayapala [2,3,4], the sound field is decomposed into spherical harmonic functions, including the scattering sound field. The sound sources can then be located using the orthogonality of the spherical harmonic functions in three-dimensional space. Li et al. studied the design and optimization of array distributions based on the genetic algorithm [5,6,7], which results in the reduction of the side lobe in the focusing spectrum. The undistorted steady focusing spectrum can be obtained by applying the quadratic constraint to the weighted coefficient using the least mean square algorithm (LMS). However, the amount of calculation required is increased for the iterative optimization algorithm, and its performance is influenced by the number of array elements, the array aperture, the sound field decomposition mode order and the source frequency, resulting in serious confusion and low resolution, rendering the accurate positioning of multiple sound sources unachievable [8].

A high-resolution and high-precision spherical reverse beamforming for sound source localization based on the inverse algorithm, which utilizes the theory of conventional spherical beamforming and the structure and sound properties of the rigid spherical array, is proposed. The performances of the array aperture, sound frequency and sound field modal order are analyzed. The simulation results show that the proposed method can be used to effectively suppress confusion and obtain higher resolution, positioning accuracy and noise suppression capabilities. The positioning performance is not affected by the frequency and the array aperture, and can therefore be applied to detect low-frequency noise sources.

## 2. Regular Spherical Array Beamforming

**Figure 1:**
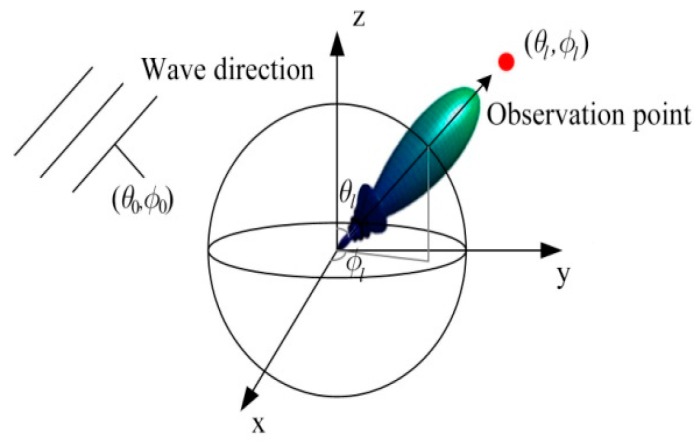
Sound source localization model for the spherical array.

The spherical array beamforming model is shown in Figure 1. We used the layout of L omni-directional microphones on the surface of a sphere of radius a. For a unit plane wave, k, incident from the direction $\left(\theta_{0},\;\varphi_{0}\right)$, the total field on the surface of the rigid sphere is [9]:

(1) $$P={\left[p_{1},p_{2},\cdots p_{l}\cdots ,p_{L}\right]}^{T}$$

where the distribution of pressure decomposed into the spherical harmonics on the sphere is given by [10]:

(2) $$p_{l}(ka,\theta_{l},\varphi_{l})=\sum_{n=0}^{N}4\pi C_{n}(ka)\sum_{m=-n}^{m=n}Y_{n}^{m*}(\theta_{s},\varphi_{s})Y_{n}^{m}(\theta_{l},\varphi_{l})$$

(3) $$C_{n}(ka)=\left\{ \begin{array}{l} i^{n}b_{n}(ka),\;\;\;\;\;\mathrm{plane}\;\mathrm{wave}\;\mathrm{source}\\ ikb_{n}(ka)h_{n}^{\left(1\right)}\left(kr_{s}\right),\;\;\mathrm{point}\;\mathrm{source} \end{array}\right.$$

where $b_{n}(ka)$ can be considered to be the mode amplitude or mode strength coefficient, and is also referred to as the sound pressure along a. It can be defined as:

(4) $$b_{n}(ka)=\left\{ \begin{array}{l} j_{n}(ka)-\frac{j_{n}^{\prime }(ka)}{h_{n}^{\prime (1)}(ka)}h_{n}^{\left(1\right)}(ka),\;\;\mathrm{rigid}\;\mathrm{sphere}\\ j_{n}(ka),\;\;\;\;\;\;\;\;\;\mathrm{open}\;\mathrm{sphere} \end{array}\right.$$

where the mode strength coefficient of the rigid sphere is the superposition of the incident field and the scattering field, $j_{n}$ is the spherical Bessel function of the order n, $Y_{n}^{m}$ is the spherical harmonics of the order n and degree m and $h_{n}^{\left(1\right)}$ is the spherical Hankel function of the first kind. The sound field should, in theory, be decomposed into a superposition of infinite order mode fields. However, the modal order is limited by the number of microphones and the analysis frequency, and the equation can be truncated to the order N [11].

In the direction (θ,φ), the weighted coefficient can be represented as:

(5) $$W={\left[w_{1},w_{2},\cdots w_{l}\cdots ,w_{L}\right]}^{T}.$$

The ideal beamforming output (F(θ,φ)) has the same properties as the Dirac function, i.e., the output peak is only in the desired direction [12,13], and in other directions it is zero:

(6) $$F\left(\theta ,\varphi \right)=\delta \left(\theta -\theta_{0},\varphi -\varphi_{0}\right).$$

Thanks to their orthonormality, the above equation can be expanded in terms of spherical harmonics:

(7) $$F\left(\theta ,\varphi \right)=2\pi \sum_{n=0}^{\infty }\sum_{m=-n}^{m=n}Y_{n}^{m*}\left(\theta_{0},\varphi_{0}\right)Y_{n}^{m}\left(\theta ,\varphi \right).$$

In [14,15], $w_{1}\left(\theta ,\varphi \right)$ was derived as followings:

(8) $$w_{l}(\theta ,\varphi )=\sum_{n=0}^{N}\frac{1}{2C_{n}(ka)}\sum_{m=-n}^{n}Y_{n}^{m}(\theta ,\varphi )Y_{n}^{m*}(\theta_{l},\varphi_{l}).$$

After complete scanning, the output of the spherical Fourier transform focused beamforming (SFTFB) method can be written as:

(9) $$F_{SFTFB}=W^{H}P.$$

The maximum value from the acoustic map gives the direction of the sound source. The SFTFB algorithm has the advantages of easier implementation and a simple calculation process, but has drawbacks such as low spatial resolution, larger sidelobes, false source identification in multiple sound source localizations and sensitivity to the environment [16]. In order to overcome these problems, one needs to utilize a correction factor and optimize the layout of the microphones [4], which increase the complexity of the calculations. In addition, because of the complexity of spherical array installations, equal angle or equidistant distributions are the most used in practice, especially for the rigid sphere, where the embedded design of the array elements makes it impossible to change their position at will. In light of this, Rafaely and Li et al. applied distortionless constraints and robustness constraints to conventional spherical beamforming [4,11], transforming the SFTFB problem into a quadratic constraint spherical focused beamforming (QCSFB) optimization problem. The following constraints were used: 

Minimum:

(10) $$W^{H}RW$$

Distortionless constraints:

(11) $$W^{H}V(\theta ,\varphi )=1$$

Robust constraint conditions:

(12) $${\left\|W\right\|}^{2}\leq T_{0}$$

where R is the cross-spectral matrix, ${\left\|W\right\|}^{2}$ is the sensitivity function and is less than the constant $T_{0}$, which makes the performance of the array more robust, and V(θ,φ) is the orientation vector, which determines the direction of array focusing [17] and can be defined as:

(13) $$V(\theta ,\varphi )=\sum_{n=0}^{N}4\pi C_{n}(ka)\sum_{m=-n}^{m=n}Y_{n}^{m*}(\theta ,\varphi )Y_{n}^{m}(\theta_{l},\varphi_{l}).$$

**Figure 2:**
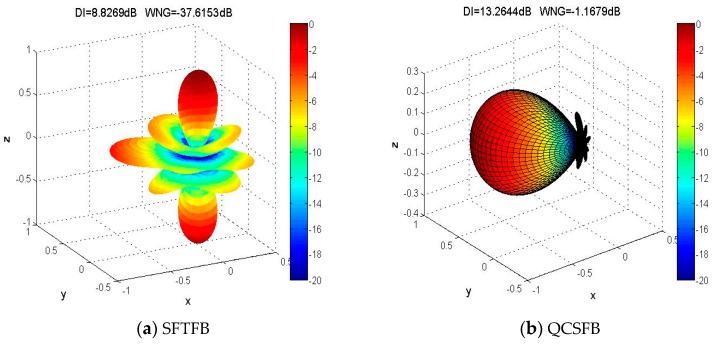
Three-dimensional beam patterns for (**a**) the spherical Fourier transform focused beamforming (SFTFB) method and (**b**) the quadratic constraint spherical focused beamforming (QCSFB) method.

In order to explain the influence of the above two methods on the beam pattern intuitively, we use an equal angle layout of 64 microphones on a rigid sphere. The three-dimensional beam pattern of order four where ka=1.8 is shown in Figure 2. The intensity of the sidelobe is almost equal to the intensity of the main lobe in the SFTFB beam pattern. This beam pattern can lead to the “confusion” phenomenon in the acoustic map, which will cause large localization errors. The directional index in Figure 2 also proves this. The sidelobe intensity is suppressed significantly in QCSFB, and the white noise gain is simultaneously improved. This makes the QCSFB method less sensitive to interference and enhances the robustness of the algorithm. However, the cost of this is that the beam width is too wide and the spatial resolution is insufficient to achieve multiple sound source localization. In addition, the Forst LMS and LMS-SP (scaled projection) algorithm is used to iteratively calculate the weighted coefficients in QCSFB, which increase the computational complexity. In light of this, a high-precision and high-resolution sound source localization method for a spherical array covering all frequencies is introduced.

## 3. Spherical Reverse Beamforming

It is assumed that there are sound sources in every beam scanning direction, including real sound sources and imaginary sound sources. All of the sound sources combined form the sound source vector $X={\left[x_{1},x_{2},\cdots x_{i}\cdots ,x_{M}\right]}^{T}$, and are collectively referred to as virtual sound sources. $x_{i}$ is the amplitude of the *i*th virtual sound source, $r_{i}$ is the distance to the array from each scan point, $\theta_{i}$ is the pitch angle and $\varphi_{i}$ is the azimuth angle. The received sound field of the *l*th microphone can be written as [18]:

(14) $$\begin{array}{ll} p_{l}(ka,\theta_{l},\varphi_{l}) & =\sum_{i=1}^{M}x_{i}\sum_{n=0}^{N}4\pi C_{n}(ka)\sum_{m=-n}^{m=n}Y_{n}^{m*}(\theta_{i},\varphi_{i})Y_{n}^{m}(\theta_{l},\varphi_{l})\\ & =\sum_{i=1}^{M}x_{i}B_{il} \end{array}$$

where

(15) $$B_{il}=\sum_{n=0}^{N}4\pi C_{n}(ka)\sum_{m=-n}^{m=n}Y_{n}^{m*}(\theta_{i},\varphi_{i})Y_{n}^{m}(\theta_{l},\varphi_{l})$$

is the sound field transfer function between the *i*th virtual source and the *l*th microphone.

The focusing output in the scanning direction (θ,φ) can be obtained from Equations (8) and (9):

(16) $$\begin{array}{ll} F(\theta ,\varphi ) & =w_{1}(\theta ,\varphi )p_{1}+w_{2}(\theta ,\varphi )p_{2}+,\cdots ,+w_{L}(\theta ,\varphi )p_{L}\\ & =\sum_{i=1}^{M}x_{i}(B_{i1}w_{1}(\theta ,\varphi )+B_{i2}w_{2}(\theta ,\varphi )+,\cdots ,+B_{iL}w_{L}(\theta ,\varphi )) \end{array}$$

Because the virtual sound sources do not contribute to the sound field, the scanning results should be same as the SFTFB results. The output at each scan point is equal to the sum of all virtual beamforms. The output matrix can be written as:

(17) $$F_{SFTFB}=GX$$

where

(18) $$G=\left[\begin{array}{c} B_{11}\omega_{1}(\theta_{1},\varphi_{1})\;B_{22}\omega_{2}(\theta_{1},\varphi_{1})\;\cdots \;B_{ML}\omega_{L}(\theta_{1},\varphi_{1})\\ B_{11}\omega_{1}(\theta_{2},\varphi_{2})\;B_{22}\omega_{2}(\theta_{2},\varphi_{2})\;\cdots \;B_{ML}\omega_{L}(\theta_{2},\varphi_{2})\\ \vdots \\ B_{11}\omega_{1}(\theta_{M},\varphi_{M})\;B_{22}\omega_{2}(\theta_{M},\varphi_{M})\;\cdots \;B_{ML}\omega_{L}(\theta_{M},\varphi_{M}) \end{array}\right].$$

The amplitude of each virtual source is:

(19) $$X=G^{-1}F_{SFTFB}$$

The real sound source can be located at the scanning point which has the largest amplitude. This is referred to as the spherical reverse focused beamforming method (SRFB). The basic idea of this method is that the actual beamforming result is a combination of all the virtual sound sources, and the amplitude of the sound sources shows the contribution of the virtual point source to the sound field, from which the position of the sound source can be determined. However, the above inverse problem is ill-posed and cannot be solved directly. An approximate solution to the problem is obtained using regularization techniques in general:

(20) $$X=G^{-1}{\left(GG^{-1}+\alpha I\right)}^{-1}F_{SFTFB}$$

where α is a regularization parameter [19,20]. The sound source information obtained based on the above formula is only an “expected” value in the sense of least squares. In order to obtain a sharpened beam to improve the sound source localization resolution, a constraint is imposed on Equation (17). In the virtual sound source vector X, the amplitude of the imaginary sound sources is zero or nearly zero, and can be thought of as a set of sparse signals. In order to make the sparsity problem easy to solve, Equation (17) is transformed into a minimization problem under the *p*-norm constraint:

(21) $$\left\{ \begin{array}{l} \min:\;E_{\left(p\right)}\;{\left\|X\right\|}_{p}\;0\leq p\leq 1\\ s.t:\;GX=F_{SFTFB} \end{array}\right..$$

The above equation is solved using the Lagrange multiplier method, and the solutions for the sound source vector (X) and Lagrange multiplier (λ) can be derived as follows:

(22) $$\left\{ \begin{array}{l} X=\Lambda^{-1}G^{H}{\left(G\Lambda^{-1}G^{H}\right)}^{-1}F_{SFTFB}\\ \lambda =-p{\left(G\Lambda^{-1}G^{H}\right)}^{-1}F_{SFTFB} \end{array}\right.$$

where

(23) $$\Lambda =diag\left({\left|X\left(i\right)\right|}^{p-2}\right)$$

is the weighted matrix. The iterative method is adopted to solve nonlinear Equation (22), in which Λ is a diagonal matrix with the results of the previous iteration on the diagonal and zeros elsewhere. As seen above, the iterative formula for solving for the sound source vector (X) is as follows:

(24) $$\left\{ \begin{array}{l} X_{}^{\left(k+1\right)}={\left(\Lambda^{\left(k\right)}\right)}^{-1}G^{H}{\left(G{\left(\Lambda^{\left(k\right)}\right)}^{-1}G^{H}+\alpha I\right)}^{-1}F_{SFTFB}\\ \lambda^{\left(k+1\right)}=-p{\left(G{\left(\Lambda^{\left(k+1\right)}\right)}^{-1}G^{H}+\alpha I\right)}^{-1}F_{SFTFB} \end{array}\right.$$

where k is the number of iterations. Similarly, the cost function for each iteration is:

(25) $$\min\Gamma^{\left(k\right)}≜\sum {\left|X^{\left(k\right)}\left(i\right)\right|}^{p}+{\left(\lambda^{\left(k\right)}\right)}^{H}\left(GX^{\left(k\right)}-F_{SFTFB}\right).\;$$

The iteration is complete when the cost function begins to increase or tends to balance. The value of *p* is not discussed in detail in this paper. For sparse reconstruction and easy solutions, the common setting is *p* = 1. Obviously, the sound pressure value of the sound source having the sparse characteristic at the focus point can be uniquely reconstructed through the above process.

## 4. Numerical Examples and Results

Target sound source localization through the SRFB method was validated using the setup of the spherical microphone array, which was described earlier. Sound sources with equal intensity were located at the positions (80°, 140°) and (140°, 250°). This setup was used to compare the low-frequency performance, the dimensionless frequency (ka=1) and the search step of the azimuth with the elevation set to 0.5° and the SNR=20 dB.

**Figure 3:**
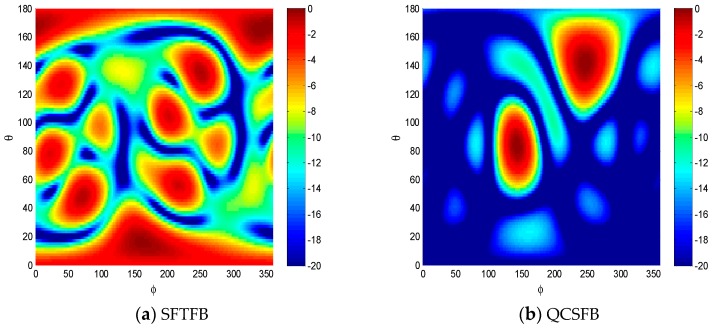

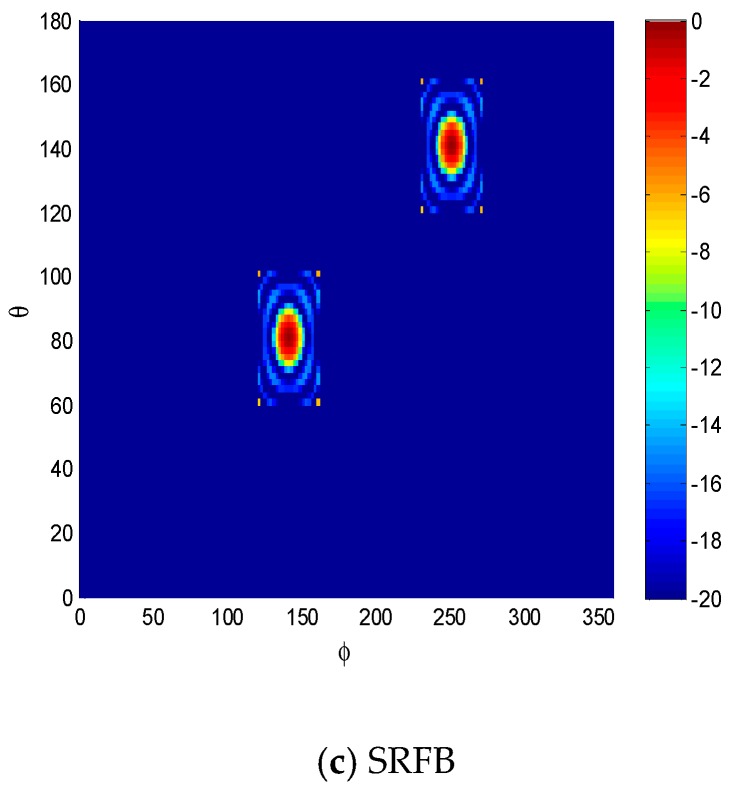
Sound sources localization simulation results for the (**a**) SFTFB, (**b**) QCSFB and (**c**) spherical reverse focused beamforming (SRFB) methods.

The sound source localization results for the three methods investigated are shown in Figure 3. At low frequencies, many false sound sources appeared when using the SFTFB method. The sidelobe could be eliminated with the implementation of QCSFB, but the spatial resolution was not high for the location accuracy of multiple sound sources. As shown in Figure 3c, the resolution can be greatly improved and the sidelobe eliminated through the implementation of the SRFB method, and the sound sources are clearly visible. The SRFB method obtained high resolution sound source location results. 

**Figure 4:**
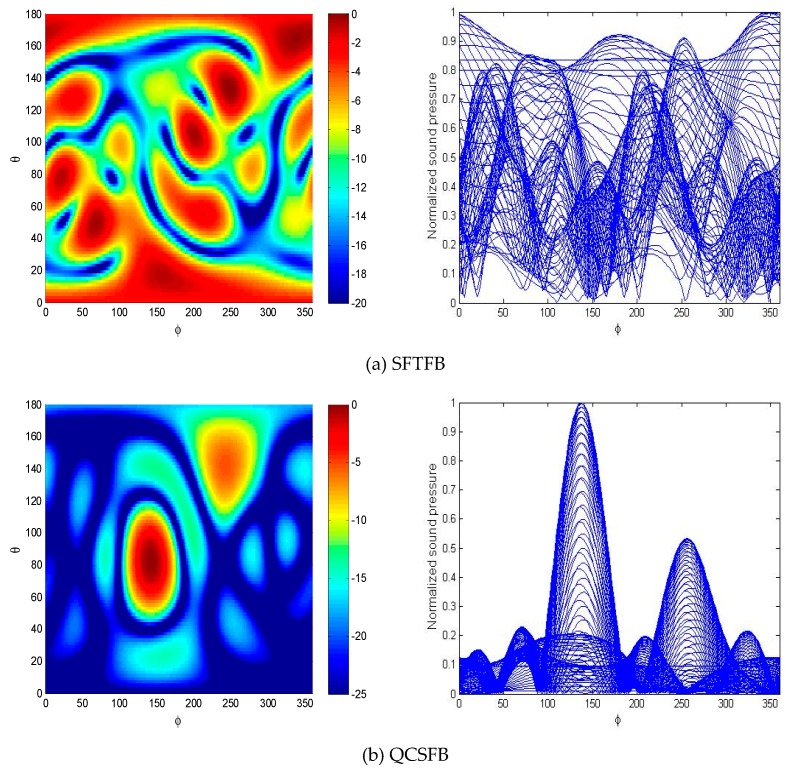

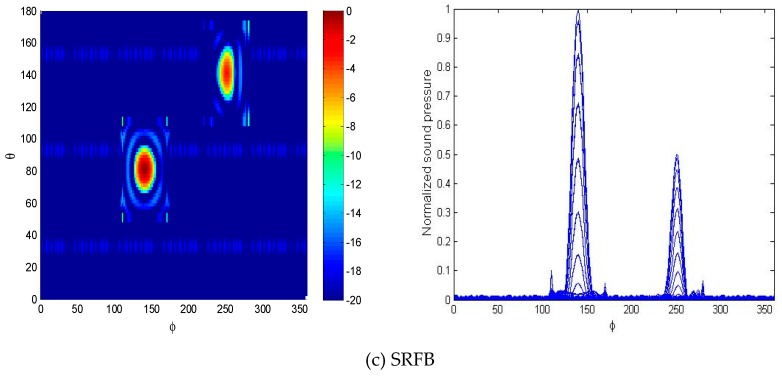
Spatial spectrum slices of two sound sources with unequal intensities obtained using the (**a**) SFTFB, (**b**) QCSFB and (**c**) SRFB methods.

The spatial localization results and spectral slices in the elevation direction of two sound sources with unequal intensities are shown in Figure 4. The sound source intensity ratio is 2:1 and the other simulation parameters are the same as above.

The same conclusion as that obtained from the first example can be obtained from Figure 4. The low-frequency sound sources were not identified by the SFTFB method because of its spatial aliasing; however, the QCSFB and SRFB methods can locate the target sources and measure the relative source intensity. The normalized sound pressure was consistent with the simulation conditions. In terms of sound source localization, the SRFB method improves the spatial resolution of the spherical array beamforming processor greatly, and has a lower background noise level, effectively overcoming the problem that low-frequency noise cannot be recognized by conventional methods.

White noise gain (WNG) is an important parameter for the characterization of the robustness of beamforming. Based on the expressions for the three algorithms given in the previous section, the equations for their respective WNGs can be written as follows:

SFTFB:

(26) $$WNG_{SFTFB}=10\log\frac{1}{{\left\|W^{H}\right\|}^{2}}$$

QCSFB:

(27) $$WNG_{QCSFB}=10\log\frac{1}{{\left\|W_{QCSFB}^{H}\right\|}^{2}}$$

SRFB:

(28) $$WNG_{SRFB}=10\log\frac{1}{{\left\|G^{-1}W^{H}\right\|}^{2}}.$$

In order to compare the performances of the three algorithms, the localization error curves, maximum sidelobe level (MSL), main beamwidth and white noise gain (WNG) were evaluated.

**Figure 5:**
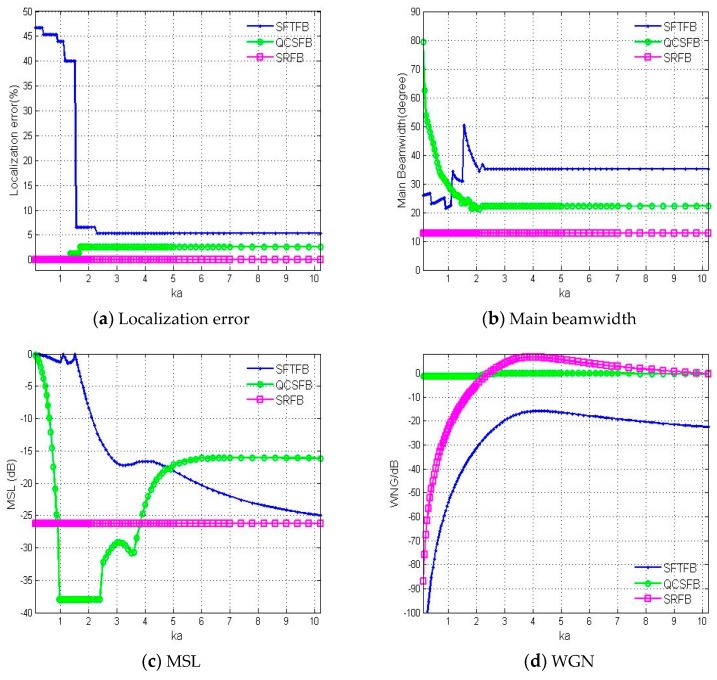
The performance of the three methods investigated based on the (**a**) localization error, (**b**) main beamwidth, (**c**) maximum sidelobe level (MSL) and (**d**) white noise gain (WNG) with respect to *ka*.

As shown in Figure 5, the localization performance was analyzed for the three methods investigated for different dimensionless parameters (*ka*). For the SFTFB method, when ka<1.5 the MSL is almost the same as the main lobe level, resulting in the presence of large amounts of false peaks and incorrect results, as seen in Figure 4a. After quadratic constraint optimization, the MSL is greatly reduced, but the main lobe width is too wide to achieve high spatial resolution and multiple sound source locations, as seen in Figure 4b. However, the sound source localization error is not affected by the parameter ka in the SRFB method, and the accuracy of sound source localization is significantly higher than those for the other two methods. Multiple sound sources can be distinguished due to the high resolution and low sidelobe level. The SRFB method can be used for high-precision beamforming over the full spectrum, as shown in Figure 4c. The WNG for the QCSFB method hardly changed with frequency because of the robust constraints, meaning QCSFB is not sensitive to interference, especially at low frequencies, which is an improvement over the other two algorithms. The robustness of the SRFB method is improved as the frequency increases.

**Figure 6:**
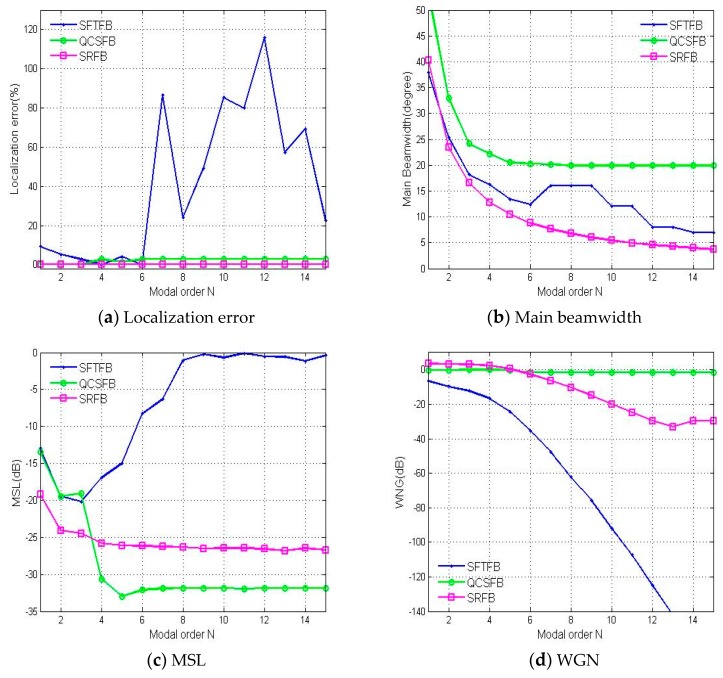
The performance of the three methods investigated based on the (**a**) localization error, (**b**) main beamwidth, (**c**) MSL and (**d**) WNG with respect to the modal order.

Figure 6 shows the influence of the modal order on localization performance. Because the three methods all performed well when ka=3∼4, as is shown in simulation results in Figure 5, we chose ka=3.6 and 64 microphones as the simulation parameters for the analysis shown in Figure 6. When N>6, the error and MSL are significantly increased for the SFTFB method, and sidelobe interference appears because the modal order is limited by the number of microphones, which is given in the literature as $L\geq {\left(N+1\right)}^{2}$ [7]. When N<6, the performance of the QCSFB method is improved with increasing modal order. Its performance is no longer influenced when N>6, and the localization error is not zero. The SRFB method can have very high spatial resolution, and its positioning error is always zero. The main beamwidth with a satisfied MSL is decreased with the increasing of the modal order and is consistently lower than other two methods. The WNGs for the SFTFB and SRFB methods decrease with increases in the modal order, with the WNG from the SFTFB method decreasing faster. It is almost impossible to output a beam with directivity, as shown in Figure 6a,c. The simulation results show that the WNG of the high-order modes can be improved by increasing the number of array elements or increasing the analysis frequency. Therefore, for practical applications, we can select the appropriate modal order according to the resolution required by the proposed method to achieve high-precision sound source localization.

## 5. The Stability of SRFB

Considering the inevitable measurement error during actual applications, two aspects of the stability of the three methods investigated were analyzed; the signal to noise ratio (SNR) and the sound field mode decomposition.

**Figure 7:**
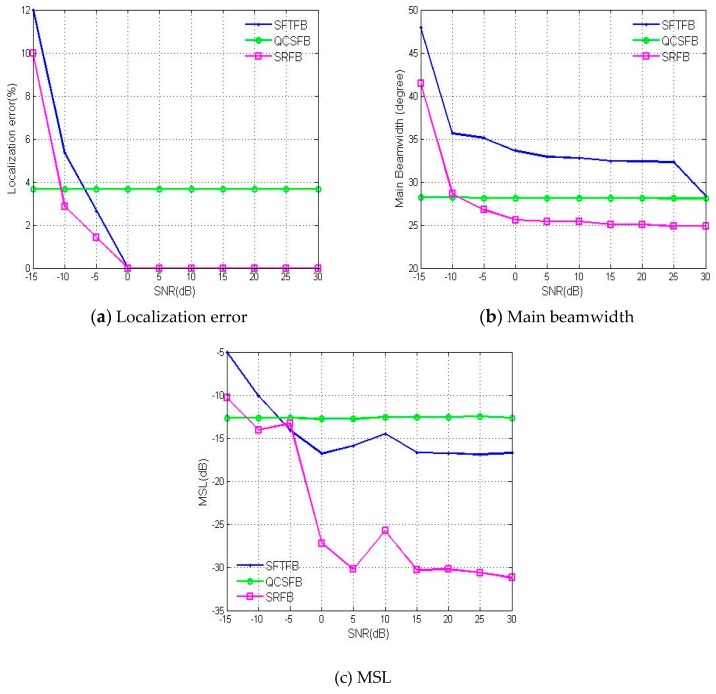
The performance of the three methods investigated based on the (**a**) localization error, (**b**) main beamwidth and (**c**) MSL with respect to the signal to noise ratio (SNR).

To begin, the performance of the three methods for different SNRs was analyzed for N=4 and ka=3.6. As shown in Figure 7, the localization performances of the three algorithms are improved with increasing SNR; however, the main beamwidth and localization error obtained using the SRFB method are superior to those obtained from the other methods. The localization error is less than 4° when the SNR is more than −10 dB. Under the condition of a low SNR, the SRFB method can achieve high-precision and high-resolution beamforming in the environment of strong background noise.

The weighted coefficients of each array element were calculated using the mode decomposition of the sound field. In this process, the modal components calculated theoretically are different from the actual sound field, which would cause a mismatch between the weighted coefficients and the array elements, and lead to a failure to locate the sound source. Therefore, it is necessary to analyze the change in performance for each method caused by modal decomposition errors.

**Figure 8:**
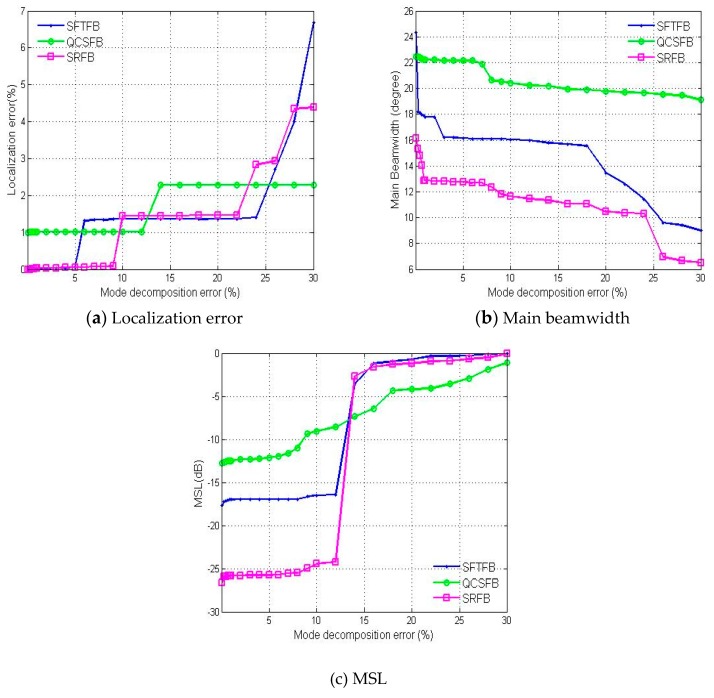
The performance of the three methods investigated based on the (**a**) localization error, (**b**) main beamwidth and (**c**) MSL with respect to the mode decomposition error.

As shown in Figure 8, the mode decomposition errors were investigated for N=4 and ka=3.6. Figure 8c indicates that the MSL is approximately 0 dB when the error is greater than 12%. Therefore, mode decomposition errors must be maintained within 12% in order to obtain meaningful results. The position error and main lobe width obtained using the three algorithms display similar trends when the mode decomposition error is increased. Under the same error conditions, the performance of spherical reverse beamforming based on the inverse method is better than that of the other two algorithms.

Through simulation analysis of the three algorithms, the stability of spherical reverse beamforming is not decreased due to the inverse process of calculation, and it can be used to obtain accurate and robust sound source localization for the full spectrum in actual measurement environments.

## 6. Conclusions

Utilizing spherical array beamforming for three-dimensional multiple sound source location, a high-precision spherical array beamforming method for sound source localization based on the inverse algorithm is proposed. The difference from previous algorithms is that regular spherical array beamforming is based on the direct radiation problem of acoustic localization. The sound field of the sphere is decomposed using spherical harmonics, and the steering vector is calculated using the orthogonality of the spherical harmonics. A beam pattern with directivity is formed by performing a weighted process. Then, the signal from a specific direction is extracted and the signals from other directions are suppressed after the beam scanning. Indeed, regular spherical array beamforming solves a scalar problem, i.e., each source is resolved separately from the others until the potential source location is identified. On the contrary, the spherical reverse beamforming method proposed in this paper is an inverse problem method. The inverse method adopts a completely different solution than the direct method. This method is based on the theory of sound field reconstruction, and assumes that the sound source surface is virtually covered with a cloud of elementary sources. Except for the actual sound source, all of the sound sources are imaginary. Then, the acoustic signals received by the spherical array are superimposed by all of the virtual elementary sources. In fact, the imaginary sound sources do not exist, that is, they do not contribute to the sound field, and therefore the focused spectrum from the actual source is the result of the focused beam output by the combined virtual sources. High-precision sound source localization can be achieved by calculating the amplitude of each virtual source. Since no beam is formed, spherical reverse beamforming aims to tackle the problem for all sources at once. The simulation results show the following: (1) The proposed method solves the problems of low resolution and grating lobes caused by the analysis frequency and array aperture in conventional spherical beamforming, leading to extremely high localization accuracy and spatial resolution. (2) Different from the constraint relationship between modal order and the number of microphones in the SFTFB algorithm, the localization performances of the QCSFB and SRFB methods increase with frequency. However, in the higher-order modes, the robustness of the SRFB method decreases slightly. (3) The localization performance of all three methods has the same downward trend with respect to the modal decomposition error. Nevertheless, the SRFB method still has the optimal performance under the same error conditions. In summary, the SRFB method has higher localization accuracy and resolution than regular spherical array beamforming, has stronger anti-interference abilities and can achieve ideal sound source localization results over the entire frequency band.
